# The somite-secreted factor Maeg promotes zebrafish embryonic angiogenesis

**DOI:** 10.18632/oncotarget.12793

**Published:** 2016-10-21

**Authors:** Xin Wang, Wei Yuan, Xueqian Wang, Jialing Qi, Yinyin Qin, Yunwei Shi, Jie Zhang, Jie Gong, Zhangji Dong, Xiaoyu Liu, Chen Sun, Renjie Chai, Ferdinand Le Noble, Dong Liu

**Affiliations:** ^1^ Co-innovation Center of Neuroregeneration, Jiangsu Key Laboratory of Neuroregeneration, Nantong University, Nantong, China; ^2^ Medical College, Nantong University, Nantong, China; ^3^ School of life science, Nantong University, Nantong, China; ^4^ Key Laboratory for Developmental Genes and Human Disease, Ministry of Education, Institute of Life Sciences, Southeast University, Nanjing, China; ^5^ Department of Cell and Developmental Biology, Karlsruhe Institute of Technology, Karlsruhe, Germany

**Keywords:** Maeg, angiogenesis, integrin, Notch, zebrafish

## Abstract

MAM and EGF containing gene (MAEG), also called Epidermal Growth Factor-like domain multiple 6 (EGFL6), belongs to the epidermal growth factor repeat superfamily. The role of Maeg in zebrafish angiogenesis remains unclear. It was demonstrated that *maeg* was dynamically expressed in zebrafish developing somite during a time window encompassing many key steps in embryonic angiogenesis. *Maeg* loss-of-function embryos showed reduced endothelial cell number and filopodia extensions of intersegmental vessels (ISVs). *Maeg* gain-of-function induced ectopic sprouting evolving into a hyperbranched and functional perfused vasculature. Mechanistically we demonstrate that Maeg promotes angiogenesis dependent on RGD domain and stimulates activation of Akt and Erk signaling *in vivo*. Loss of Maeg or Itgb1, augmented expression of Notch receptors, and inhibiting Notch signaling or Dll4 partially rescued angiogenic phenotypes suggesting that Notch acts downstream of Itgb1. We conclude that Maeg acts as a positive regulator of angiogenic cell behavior and formation of functional vessels.

## INTRODUCTION

A number of secreted factors produced by surrounding cells and tissues regulate angiogenesis through mediating endothelial cells (ECs) differentiation, proliferation, and migration [1-3]. The epidermal growth factor (EGF) repeats is a widely distributed module in many different proteins in single or multiple copies that was first described in 1972 within the EGF protein [4, 5]. Interestingly, EGF-like proteins such as Betacellulin (BTC) [6], Heparin-binding EGF-like growth factor (HB-EGF) [7-10], and EGFL7 [11-17] have been demonstrated to play vital roles in angiogenesis and endothelial cell behaviors. MAEG (MAM and EGF containing gene), also named EGFL6, was first identified in 1999 as an EGF repeat-containing protein [18]. Adipose secreted MAEG has been proved to promote proliferation of adipose tissue-derived stromal vascular cells [19]. Moreover, that osteoblastic-like cells express MAEG that is capable of promoting endothelial cell migration and angiogenesis via ERK activation [20]. However, the functional analysis of Maeg involved in embryonic vascular development of vertebrate *in vivo* is thus far lacking.

The vascular network develops in a conserved manner in all vertebrates. The zebrafish (Danio rerio) model system offers distinct advantages for *in vivo* studies of the vascular development [21]. In the present study, we analyzed the expression dynamics of Maeg in zebrafish developing somite by using whole-mount *in situ* hybridization, and immunostaining. Then we examined whether zebrafish *maeg* regulates angiogenesis *in vivo* through loss- and gain-of-function analysis. Further more, we investigated the potential mechanism underlying *maeg* regulating zebrafish embryonic angiogenesis.

## RESULTS

### Maeg dynamically expressed in zebrafish developing somite

**Figure 1:**
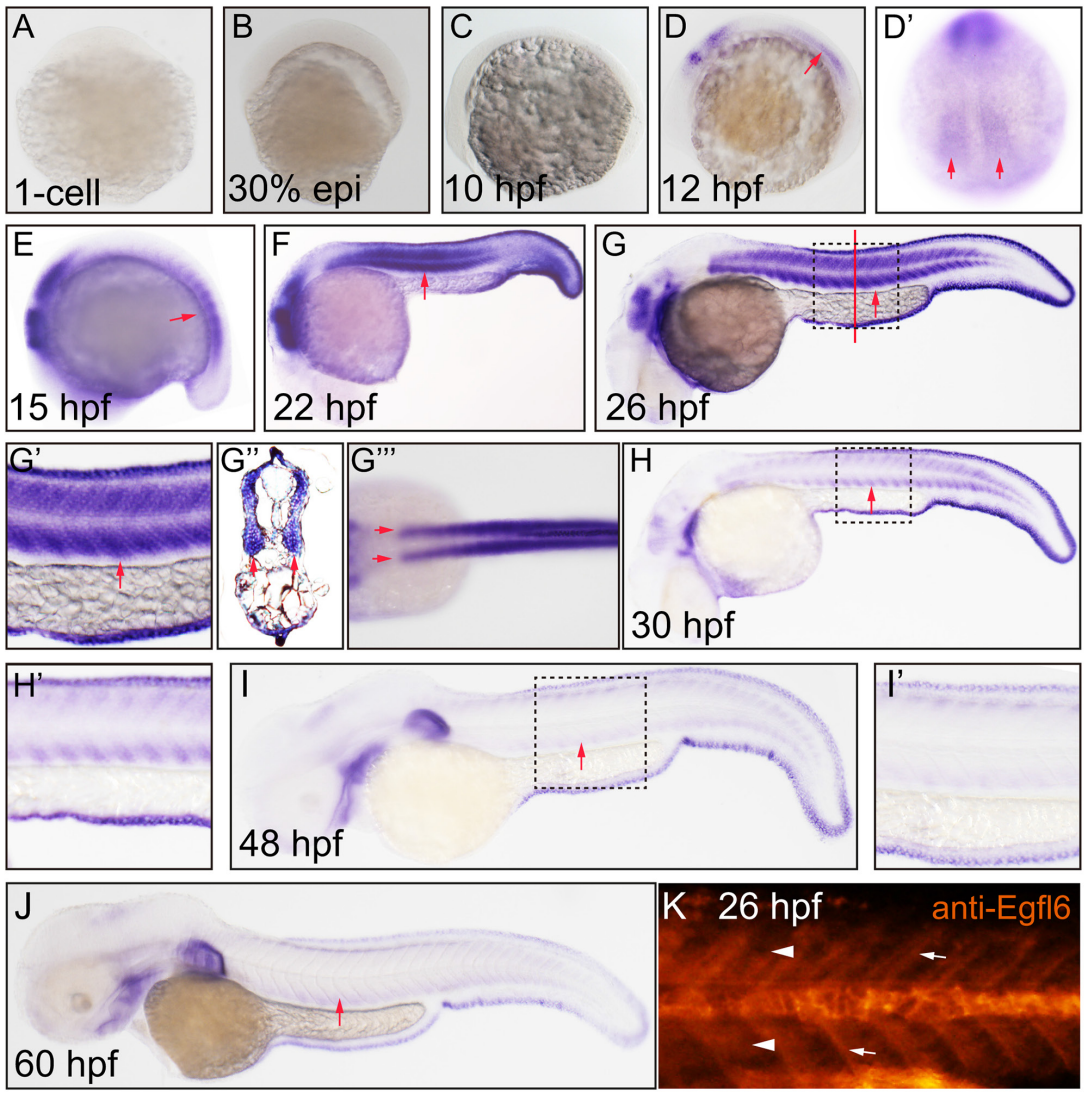
Maeg dynamically expressed in zebrafish developing somite Expression of *maeg* was analyzed by whole mount *in situ* hybridization and whole mount antibody staining. **A.** 1-cell, lateral view, no staining. **B.** 30% epiboly, lateral view, no staining. **C.** 10 hpf, lateral view, no staining. **D.** 12 hpf, lateral view, arrow indicates somite. **D’.** 12 hpf, lateral view, arrows indicate somite. **E.** 15 hpf, lateral view, arrow indicates somite. **F.** 22 hpf, lateral view, arrow indicates somite. **G.** 26 hpf, lateral view, arrow indicates somite, square in dash line indicates the magnified region in **G’.** red line indicates the section position **G’’, G’’’.** 26 hpf, dorsal view, arrows indicate myotomes. **H.** 30 hpf, lateral view, arrow indicates somite, square in dash line indicates the magnified region in **H’, I.** 48 hpf, lateral view, arrow indicates somite, square in dash line indicates the magnified region in **I’, J.** 60 hpf, lateral view, arrow indicates somite. **K.** 26 hpf, lateral view, arrowheads indicate somites, arrows indicate somite borders.

To investigate the expression dynamics of Maeg in zebrafish developing somite, we did the detailed whole amount *in situ* hybridization (WISH) analysis using digoxigenin-labeled antisense probes. Consistent with previous report [22], the hybridization signal was apparently shown in myotome at 12 hpf and increasingly maintained at 15 hpf (Figure 1A-1E). The somital expression of *maeg* reached its peak at around 22~26 hpf and decreased hereafter (Figure 1F-1G’’’). The hybridization signal of *maeg* in somite became very weak at 30 hpf and barely detectable at 48 and 60 hpf (Figure 1H-1J). Taken together, *maeg* dynamically expressed in zebrafish developing somite during a time window encompassing many key steps in embryonic angiogenesis. In addition, the expression dynamics of *maeg* in hindbrain was similar with that in somites (Figure 1A-1J). Whole-mount immunostaining analysis of Maeg in Wild type (WT) embryos showed that Maeg protein was present in somites and highly accumulated in somite borders (Figure 1K).

### Establishment of *maeg* knockout mutant line by TALEN

**Figure 2:**
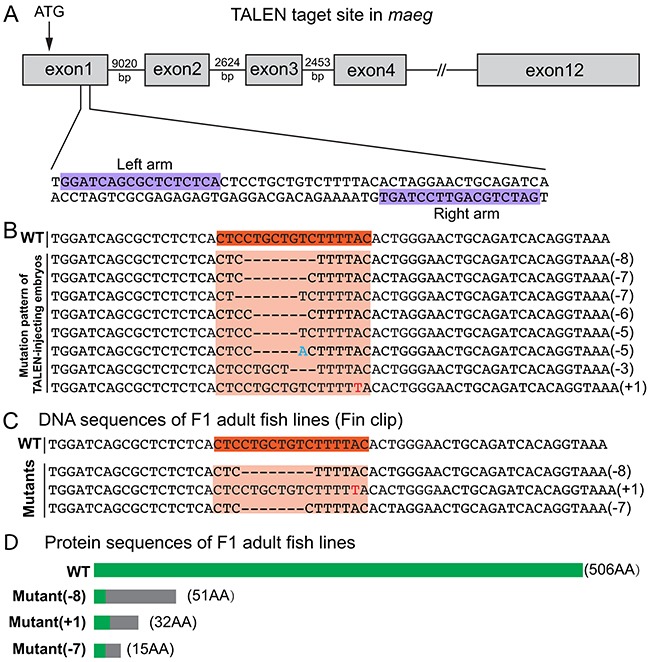
Generation of zebrafish *maeg* mutant using TALENs **A.** Schematic diagram showing TALEN targeting site on the first exon of *maeg* gene. Starting codon (ATG) site is indicated by arrow. The left and right TALEN targeting sites are highlighted in purple. **B.** Mutation pattern of TALEN-injecting embryos. Numbers in the brackets show the number of nucleotides were deleted (−) or inserted (+). Inserted nucleotide is in red. WT, wild-type. **C.** Three heritable mutants were identified by screening. F0 founder fish were out-crossed with WT fish to produce F1, and the DNA extracted from tail fins of F1 adults were used for identification of heritable mutants by sequencing. **D.** Schematic diagram showing the predicted proteins encoded by the three mutated alleles. The mutants are reading frameshift mutations that result in truncated proteins. The gray rectangles indicate the wrong coded amino acid sequences.

To do the loss of function analysis on *maeg* gene in zebrafish, TALEN was utilized to establish *maeg* knockout mutant line. To ensure complete disruption of functional proteins, we chose the TALEN target sites near the translation start codon in the first exon of zebrafish *maeg* (Figure 2A, Supplementary Figure S1A). Then we built two constructs containing left and right arms of *maeg* TALEN respectively. The *in vitro* synthesized mRNAs of the two arms were microinjected into the cytoplasm of 1-cell stage zebrafish embryos. TALEN *in vivo* activity was analyzed by HRMA. The TALEN proteins turned out to efficiently induce mutations in the targeting site (Supplementary Figure S1B). To evaluate the mutagenesis frequency, a 150-bp genomic DNA fragment containing the target site was PCR amplified from 32 TALEN injected embryos at 24 hpf and sequenced. Sequence analysis revealed that the mutated rate of *maeg* alleles was 43.8% (14/32) and 8 types of mutations (Figure 2B). The remaining siblings of these F0 embryos were raised to adulthood. The healthy F0 founders carrying somatic mutations were out-crossed with wild type fish to obtain F1 offspring. Among the adult F1 offspring, 3 types of mutations were identified via PCR amplification and sequencing with fin-clipped genomic DNAs (Figure 2C, S1C). The mutated alleles included an 8-bp deletion, a 7-bp deletion and a 1-bp insertion, which all result in reading frame shift and premature translation termination (Figure 2C). The Maeg protein ablation in 7-bp deletion line was confirmed by western blot analysis (Supplementary Figure S2C). For the subsequent experiments, the 7-bp deletion mutant line was selected to outcross with *Tg(fli1a:nEGFP)* and *Tg(kdrl:EGFP)* transgenic lines for studying angiogenesis. Homozygous *maeg* mutant individuals (*maeg*^−/−^) were obtained in F3 offspring.

### Loss of Maeg impairs intersegmental vessels branching angiogenesis

**Figure 3:**
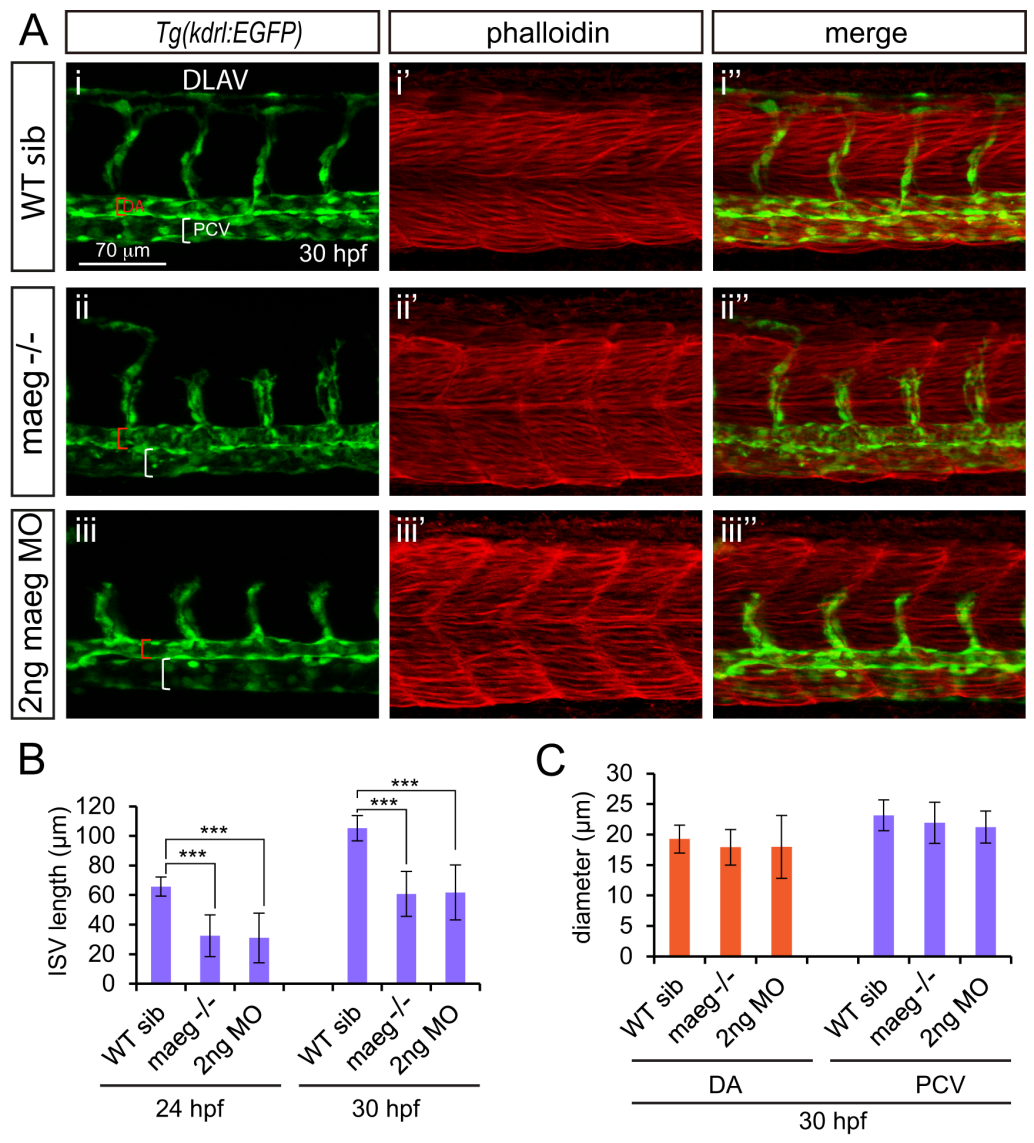
*maeg* loss of function results in the blood vessel morphogenesis defects in zebrafish embryos **A.** Confocal imaging analysis of trunk vascular and somital morphology in WT, *maeg*^−/−^ and *maeg* morphants *Tg(kdrl:EGFP)* embryos at 30 hpf. Red and white square brackets indicate the lumen of the DA and PCV, respectively. DA, dorsal aorta; PCV, posterior cardinal vein; and DLAV, dorsal longitudinal anastomotic vessel. **B.** The statistics of ISV length in WT, *maeg*^−/−^ and *maeg* morphants. One-Way ANOVA; ***,*P*<0.001. **C.** The statistics of DA and PCV lumen size at 30 hpf. Error bars indicate stdev.

To investigate the role of Maeg in embryonic angiogenesis, we examined vascular development of *maeg*^−/−^ *Tg(kdrl:EGFP)* zebrafish embryo at different stages using *in vivo* confocal imaging. In the *maeg* mutants, although the position of the initial sprout formation was not affected (Figure 3A, S2B), at 24 hpf the length of intersegmental vessel (ISV) is significantly shorter than that of control (Figure 3B, S2B). At 30 hpf stage the ISVs grew only halfway through their ventral trajectory and usually failed to cross the horizontal myoseptum (Figure 3A, 3B). As a consequence, formation of the dorsal lateral anastomotic vessel (DLAV) was severely disturbed (Figure 3A). Loss of *maeg* did not apparently affect the diameter of axial vessels, including DA and posterior cardinal vein (PCV), which are formed via vasculogenesis (Figure 3C). To confirm the angiogenic phenotype of *maeg* mutants, we knocked down *maeg* using two mopholinos (MO, a mopholino for blocking translation and a morpholino for modifying pre-mRNA splicing), whose specificity were validated by Western Blot and RT-PCR analysis as previous reported [22]. Confocal imaging analysis revealed that morpholino-mediated knockdown of *maeg* phenocopied the angiogenic defects of *maeg* mutants (Figure 3A, 3B, 3C).

Due to the localization of Maeg in somites and the borders, we evaluated weather the angiogenesis defects in *maeg* mutants were caused by the abnormalities of somites and the borders. Through the phalloidin staining, immunostaining and microscope imaging analysis, we did not observe the obvious defects of somites and the borders in *maeg* deficiency embryos (Figure 3A, S3A-S3D). We also examined weather *maeg* is involved in embryonic hindbrain development in *Tg(huC:EGFP)* embryos. Confocal imaging analysis of the fluorescence labeled neurons in *Tg(huC:EGFP)* embryos revealed normal hindbrain development in *maeg* loss of function zebrafish (Supplementary Figure S3E, S3F).

### Maeg overexpression causes excessive branching

**Figure 4:**
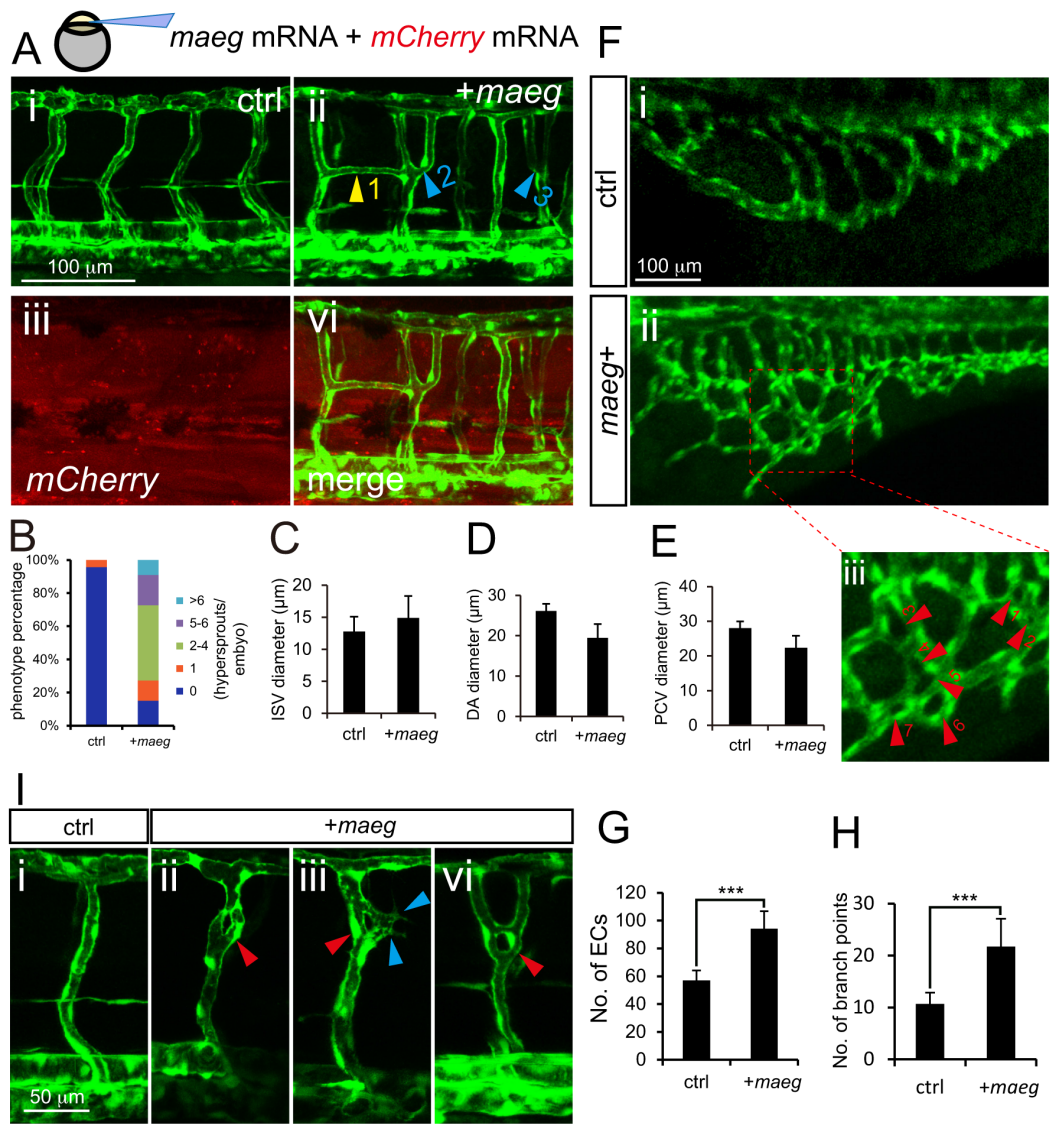
Maeg overexpression causes excessive branching **A.** Confocal imaging analysis of trunk vascular morphology in control and *maeg*/*mCherry* mRNA mixture injected *Tg(kdrl:EGFP)* embryos at 48 hpf. Yellow arrowhead indicates the aberrant vessel connected two adjacent ISVs. Blue arrowheads indicate Y-shaped ISVs. **B.** The statistics of hyperbranching sprouts in *maeg* up-regulated embryos. **C-E.** The statistics of ISV, DA and PCV lumen size at 48 hpf. Error bars indicate s.e.m. **F.** Morphology of subintestinal vessel (SIVs) in 72 hpf *Tg(fli1a:EGFP)* embryos injected with control mRNA or *maeg* mRNA. **G.** The statistics of branch point number. Student's t-test; ***,*P*<0.001. **H.** Quantification of ECs nuclei number in SIVs. Student's t-test; ***,*P*<0.001. **I.** Confocal imaging analysis of ISVs morphology in control and *maeg* mRNA injected *Tg(kdrl:EGFP)* embryos at 48 hpf. Red arrowheads indicate knot-like structures. Blue arrowheads indicate angiogenic sprouts.

To further determine the role of *maeg* in angiogenesis, we did the *maeg* gain of function analysis in zebrafish embryos. We observed the excessive branching of ISVs in *maeg* mRNA injected embryos (Figure 4A). In control 48 hpf embryos, the paralleled ISVs from DA or PCV are separated single tubes and directly connected to the DLAV (Figure 4A-4i). In *maeg* up-regulated embryos, there were usually two sorts of phenotypes of excessively branched ISV. One of those is ISV from the axial vessels gave rise to two sprouts and connected with DLAV respectively, forming Y-shaped structure (Figure 4A-4ii). And another is that one of the ISVs branched an additional sprout from its middle position to connect with adjacent one, forming H-shaped structure (Figure 4A-4ii; Supplementary Figure S4A). More than 80% *maeg* over expression embryos had no less than one excessively branched ISV (Figure 4B). We also noted these aberrant branching points mostly happened in the dorsal half of the ISVs. In some cases, these phenotypes combined or repeated to form more complex structures (Figure 4A-4ii, Supplementary Figure S4B, S4C). These aberrant vessel connections formed a lumen and perfused with blood flow (Figure 4A). The diameter of ISV in *maeg* gain of function embryos is slightly larger than that in control embryos (Figure 4C). And over expression of *maeg* did not significantly affect the diameters of axial vessels (Figure 4D, 4E). Furthermore we checked the identity of those hyperbranched ISVs and found around 75% of them were arteries. In addition we observed significant hyperbranching in subintestinal vessels (SIV) (Figure 4F). Overexpression of *maeg* resulted in the SIV became a much more complex structure, with the ECs numbers and branch points increased dramatically (Figure 4F, 4G, 4H). Interestingly, knot-like structures were observed in some of the ISVs in *maeg* gain of function embryos (Figure 4I). Around 28%(5 of 18) of the *maeg* over expression embryos we analyzed exhibited this sort of phenotype. However, we did not find this structure in control embryos. The knot-like structure was also shown to continue branching and form more complex structures (Figure 4I-4iii, -vi).

### Maeg regulates the endothelial tip cell behaviors

**Figure 5:**
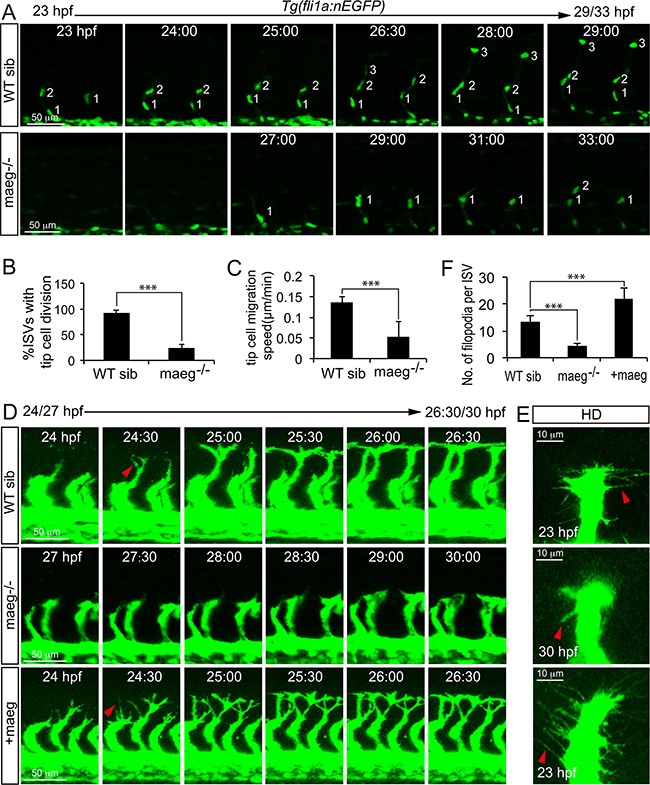
Maeg regulates ISV tip cell behaviors **A.** Still images from *in vivo* time-lapse imaging analysis of WT and *maeg*^−/−^*Tg(fli1a:nEGFP)* embryos. Time (hpf) is noted in the top. Nuclei of ISVs are numbered. **B.** Percentage of ISVs with tip cell division in control embryos and *maeg*^−/−^ embryos. Student's t-test; ***,*P*<0.001. **C.** Migration speed of ISV tip cells. Mann Whitney U-test; ***,*P*<0.001. **D.** Still images from *in vivo* time-lapse imaging analysis of ISV tip cell filopodia in *Tg(kdrl:EGFP)* embryos. Time (hpf) is noted in the bottom. Red arrowheads indicate filopodia extensions. **E.** Confocal imaging analysis of ISV tip cell filopodia in *Tg(kdrl:EGFP)* embryos with HD detection setting. Red arrowheads indicate filopodia extensions. **F.** ISV tip cell filopodia number in per ISVs. One-Way ANOVA; ***, *P*<0.001.

Since *maeg* was required for ISVs outgrowth, we reasoned that it might play a role in governing endothelial tip cell behaviors. To determine if this was the case and study the cellular mechanisms underlying the branching angiogenic defect of ISVs caused by loss of *maeg*, we analyzed the effects of *maeg* on tip cell behaviors in zebrafish embryos. Firstly we examined the tip cell proliferation, and migration using *in vivo* time-lapse imaging of *Tg(fli1a:nEGFP)* embryos, in which EGFP accumulates in nucleus in ECs [23]. In control embryos, the endothelial tip cells of ISV initiated from DA at around 20 hpf and migrated towards the dorsal direction along their trajectory, as described in previous work [16]. Once arriving at the horizontal myoseptum, tip cells in most ISVs underwent proliferation into 2 ECS (Figure 5A), and one of the daughter cell continuously migrate to the dorsal roof to form DLAV (Figure 5A). In the absence of *maeg*, the ISV tip cells migrated from DA later than the stage of control (Figure 5A). Furthermore, the subsequent migration of tip cells from the horizontal myoseptum to the DLAV was slower than that in the control embryos (Figure 5C). Some of the tip cells still stopped at the horizontal myoseptum in 33 hpf *maeg* mutants (Figure 5A). In addition, half of the tip cells failed to proliferate (Figure 5B). These observations suggest that *maeg* regulates both migration and proliferation of tip cells during ISV branching angiogenesis. Endothelial tip cells extend dynamic filopodia to sense the surroundings and lead the outgrowth of capillaries. Tip cell filopodia extensions of ISVs appeared shorter and less in *maeg* mutants (Figure 5D-5F) compared with that of controls (Figure 5D-5F). In contrast, endothelial tip cell filopodia extensions of vessel sprouts appeared more abundant in *maeg* gain of function embryos (Figure 5D-5F). In addition, the *maeg* gain of function embryos showed the ectopic branching angiogenic behaviors in the ISV and DLAV (Supplementary Figure S5). These results are in agreement with the branching angiogenic defects in *maeg* loss and gain of function embryos.

### Maeg promotes angiogenesis dependent on RGD domain and mediates activation of Akt/ERK signaling *in vivo*

**Figure 6:**
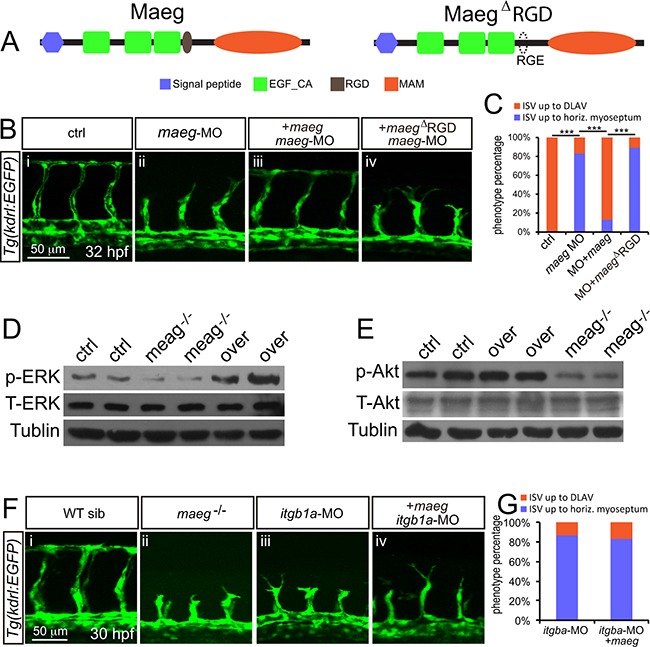
Maeg regulates angiogenesis dependent on RGD domain **A.** The diagram of Maeg protein with RGD domain and Maeg protein with RGD domain mutated to RGE domain (Maeg^ΔRGD^). **B.** Confocal images of ISVs in control embryos, *maeg* morphants, *maeg* morphants treated with DAPT, and *maeg* morphants coinjected with *dll4* MO using *Tg(kdrl:EGFP)* transgenic embryos. **C.** Percentage of embryos with ISV defect in each group. χ^2^ test; ***, *P*<0.001. **D, E.** Phosphorylation levels of ERK and Akt determined by Western blot analysis in *maeg* loss- and gain-of-function embryos. **F.** Confocal images of ISVs in control embryos, *maeg* mutants, *itgb1a* morphants, and *itgb1a* morphants coinjected with *maeg* mRNA using *Tg(kdrl:EGFP)* transgenic embryos. **G.** Percentage of embryos with ISV defect in each group.

Maeg was reported to contain a RGD domain that binds to its receptor integrins [18, 24]. Through bioinformatical analysis we proved zebrafish Maeg contain a conserved RGD domain as well [22]. To determine whether Maeg regulates angiogenesis through interaction with integrins, we examined the function of RGD domain on the vascular phenotype of *maeg*-deficency zebrafish embryos. Coinjection of an ATG-MO-1 resistant form of *maeg* mRNA significantly reduced the ratio of ISV branching defect (Figure 6B-6iii, 6C). However coinjection of an ATG-MO-1 resistant form of *maeg* mRNA with RDG domain mutated to RGE failed to rescue the ISV length (Figure 6A, B-iv, 6C). These results suggest the *maeg* regulates embryonic angiogenesis dependent on RGD domain through integrins. Ligation of integrins triggers a large variety of signal transduction events including PI3K/Akt and MEK/ERK [25]. The activation of ERK and Akt pathway is necessary for several key EC functions, including proliferation, migration, survival, and vascular tone [26, 27]. In addition, ERK pathway is strongly activated by Maeg in SVEC (a simian virus 40-transformed mouse microvascular endothelial cell line) [20]. Therefore we examined the effect of Maeg on the activation of ERK and Akt pathways *in vivo* by Western Blot using specific phosphorylated antibodies to p-ERK and p-Akt. It was shown that the p-ERK and p-Akt was down regulated in *maeg* mutants (Figure 6D, 6E). Subsequently, we observed that blocking the function of MEK with specific inhibitor U0126 or PD98059 treatment resulted in sprouting angiogenesis defects, as previous reported [28], with reduced length of ISV and decreased number of ECs (Supplementary Figure S6). These phenotypes are reminiscent of those in *maeg* deficiency embryos. Taken together, these findings indicate ERK is involved in Maeg mediated angiogenesis. Integrin β1 (Itgb1) was reported to be one of the receptor for Maeg recognizing the RGD motif [29]. Then we did a series of experiments to testify weather integrin β1 was the receptor through which Maeg regulates angiogenesis in zebrafish embryos. Based on the previous studies, there are four *itgb1* isoforms in zebrafish, including *itgb1a*, *itgb1b*, *itgb1b.1*, and *itgb1b.2* [30, 31]. We isolated GFP+ cells from *Tg(kdrl:EGFP)* transgenic embryos at 24 hpf by fluorescence-activated cell sorting (FACs) (Supplementary Figure S7A). The purity of these sorted cells was validated by FAC resorting and Taqman PCR analysis of marker genes of the ECs (Supplementary Figure S7B, S7C). The expression levels of *itgb1* isoforms were determined by RT-PCR Assay (Supplementary Figure S7D), showing *itgb1a* was expressed in ECs of 24 hpf zebrafish embryos (Supplementary Figure S7D). These results were confirmed by whole-mount *in situ* hybridization data (Supplementary Figure S7E-S7H). Subsequently, we knockdown the *itgb1a* using a translation blocking MO in *Tg(kdrl:EGFP)* zebrafish embryos and observed that *itgb1a* morphants phenocopied the angiogenic defects of *maeg* mutants (Figure F6F-iii). In addition, overexpression of *maeg* in *itgb1a* deficiency embryos did not rescue the phenotype of *itgb1a* morphants (Figure F6F-iv, 6G). Taken together, these data suggested that *maeg* promotes angiogenesis in zebrafish embryos through Integrin β1.

### Notch signaling in *maeg* deficiency embryos

**Figure 7:**
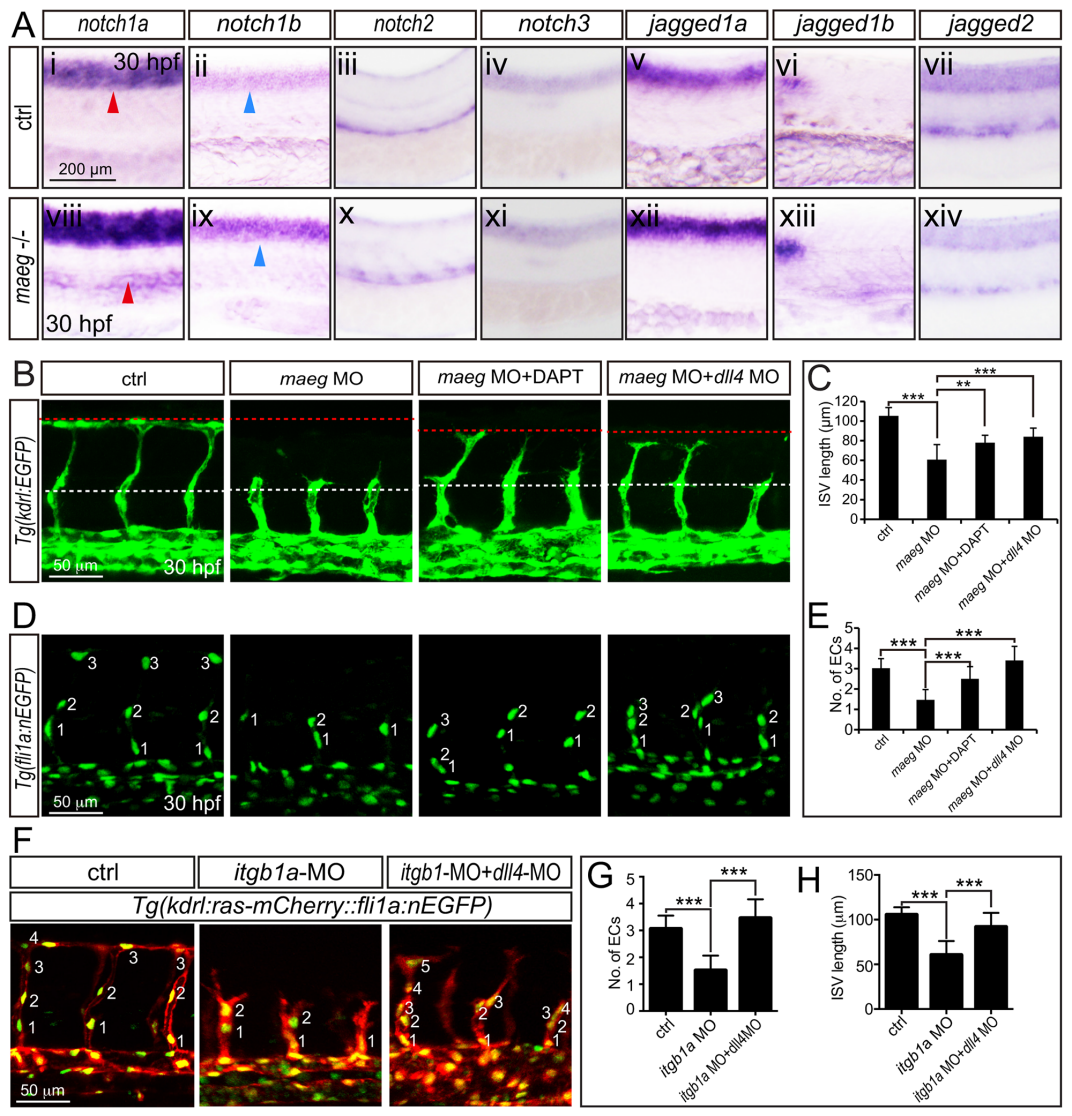
The phenotype of *maeg* and *itgb1* loss-of-function involves Notch signaling **A.** Whole mount in situ hybridization analysis of zebrafish embryos using antisense *notch1a*, *notch1b*, *notch2*, *notch3*, *jag1a*, *jag1b*, and *jag2b* probes. 30 hpf, lateral view. Blue arrowheads indicate the notochord position. Red arrowheads indicate the trunk vessel position. **B.** Confocal images of ISVs in control embryos, *maeg* morphants, *maeg* morphants treated with DAPT, and *maeg* morphants coinjected with *dll4* MO using *Tg(kdrl:EGFP)* transgenic embryos. The red and white dash lines indicate the position of dorsal roof and horizontal myoseptum respectively. **C.** The statistics of ISV length in 30 hpf control embryos, *maeg* morphants, *maeg* morphants treated with DAPT, and *maeg* morphants coinjected with *dll4* MO. One-Way ANOVA; ***,*P*<0.001. **D.** Confocal imaging analysis of endothelial numbers of ISVs in 30 hpf control embryos, *maeg* morphants, *maeg* morphants treated with DAPT, and *maeg* morphants coinjected with *dll4* MO using *Tg(fli1a:EGFP)* transgenic embryos. Nuclei of ISVs are numbered. **E.** Quantification of ECs nuclei number in ISV. Measurements were made from three adjacent ISVs (over yolk) per embryo from 3 independent experiments. One-Way ANOVA; ***,*P*<0.001. **F.** Confocal images of ISVs in control embryos, *itgb1a* morphants, and *itgb1a* morphants coinjected with *dll4* MO using *Tg(kdrl:ras-mCherry::fli1a:nEGFP)* transgenic line at 30 hpf. **G.** Quantification of ECs nuclei number in ISV. Measurements were made from three adjacent ISVs (over yolk) per embryo from 3 independent experiments. One-Way ANOVA; ***,*P*<0.001. **H.** The statistics of ISV length. One-Way ANOVA; ***,*P*<0.001.

Egf-like family member has been implicated in the modulation of Notch signaling [16, 32, 33]. The Notch signaling pathway has been well documented to be involved in angiogenic cell behavior in ISVs [34, 35]. Therefor we hypothesized *maeg* promoted zebrafish embryonic branching angiogenesis involving Notch signaling. Then we examined the expression level of Notch receptors and ligands in *maeg* deficiency embryos. Our data sets showed that the expression level of the *notch1a* and *notch1b* was elevated (Figure 7A-7viii, -ix). These data suggest that *maeg* inhibits Notch signaling. If the up-regulation of Notch signaling is responsible for the ISV angiogenic defects in *maeg* deficiency embryos, reducing Notch signaling level will restore the angiogenic potential. To test this hypothesis, we treated the *maeg* morphants with the Notch γ-secretase inhibitor DAPT and knock down *dll4* expression by MO injection. We found that inhibition of Notch signaling did not completely rescue the ISV length of *maeg* deficiency embryos (Figure 7B, 7D). But it restored the endothelial cell number (Figure 7C, 7E). Thus, loss of *maeg* is associated with the increase of Notch, whereas inhibition of Notch is not sufficient to rescue the vascular branching phenotype in *maeg* loss-of-function embryos. Furthermore, we did a series of experiments to testify whether the Notch signaling is downstream of Itgb1. It was revealed that *notch1a* and *deltaC* were increased in *itgb1a* morphants (Supplementary Figure S8A). DAPT treatment and down-regulation of *dll4* partially rescued the ISV branching defects caused by loss of *itgb1a* (Figure 7F-7H, S8B). These data suggest that Notch signaling acts downstream of Itgb1.

## DISCUSSION

**Figure 8:**
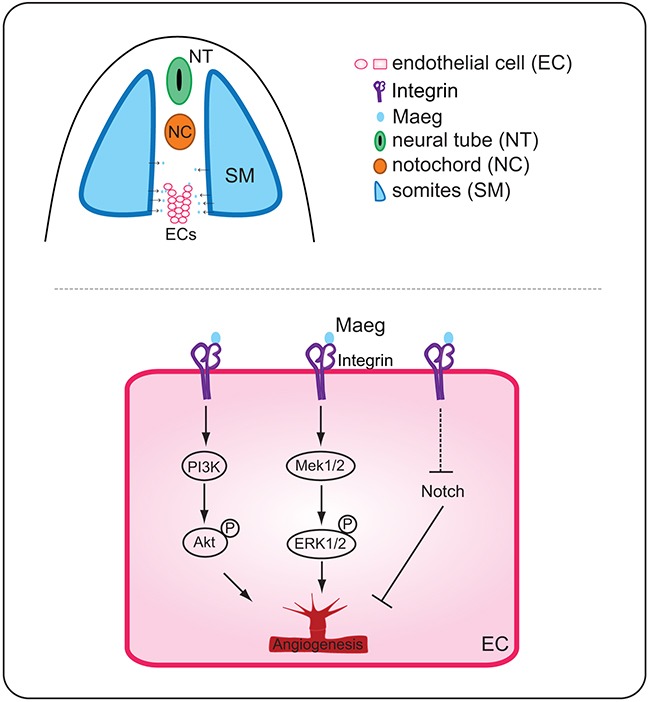
A working model for the function of Maeg in angiogenesis Binding of Maeg to their receptors on ECs leads to activation of the PI3K/Akt and Mek/ERK signaling pathways, which are involved in angiogenesis. Maeg/Itgb1 negatively regulate Notch signaling, which inhibits angiogenesis.

EGF-like proteins have been suggested to play a variety of roles in angiogenesis and endothelial cell behaviors. BTC, a member of the EGF family, induces angiogenesis through activation of mitogen-activated protein kinase (MAPK) and phosphatidylinositol 3′-kinase (PI3K) in human umbilical vein endothelial cells (HUVECs) [6]. In addition, using the mouse Matrigel plug assay BTC was proved to be capable of promoting angiogenesis *in vivo*[6]. HB-EGF belongs to the EGF superfamily of ligands. HB-EGF-induced HUVEC migration and capillary tube formation were dependent upon activation of PI3K and MAPK signaling pathways but were independent of the endothelial cell behaviors induced by VEGF [7-10]. Most secreted angiogenic signaling molecules are mainly produced by non-endothelial cell types. In contrast, EGFL7 is a unique secreted angiogenic factor because it is almost exclusively expressed by and acts on endothelial cells. It is important for regulating tubulogenesis in zebrafish and for controlling vascular patterning and integrity in mice [11, 17]. Its function in blood vessel development is mediated, at least in part, through modulation of Notch signaling and Akt/ERK activation [11-16, 36]. Currently, *maeg* was demonstrated to promote angiogenesis dependent on RGD domain and mediates activation of Akt/ERK signaling *in vivo* (Figure 8). Zebrafish Maeg and the other several Egf-like proteins share the similar downstream signaling pathway, through which they regulate angiogenesis. In support, Nichol et al showed that 25% Tie2-Egfl7 transgenic mice exhibited knot-like structure vessels [16]. Similarly, knot-like structures were also observed in ISVs of *maeg* gain-of-function embryos. The ERK pathway is activated by MAEG whereas Akt remains constant in SEVC (a simian virus 40-transformed mouse microvascular endothelial cell line) cells [20]. This discrepancy is possibly due to the different system was used.

Altered Notch signaling affects several aspects of angiogenesis, including angiogenic cell behavior and tip cell differentiation in segmental sprouts [34, 37]. Inactivation of *maeg* in zebrafish embryos impaired ISV out growth and tip cell behaviors that is reminiscent of Notch activation. In Maeg mutants, Notch signaling was upregulated and this result was consistent with previous studies that Egf-like family members have been implicated in the modulation of Notch signaling. Nueda et al revealed that the EGF-like protein DLK1 Inhibits Notch signaling and potentiates adipogenesis of mesenchymal cells [33]. It was also shown that EGFL7 interacts with receptors of the Notch family and acts as an antagonist of the Notch signaling pathway in cultured neural stem cells [32]. In particular, Nichol et al showed that EGFL7 regulates blood vessel development, at least in part, by modulation of Notch signaling [16]. EGFL7 overexpression in the postnatal retina and in primary endothelial cells reduces Notch target gene expression and induces a subtle hyperangiogenic response, similar to what has been observed when Notch signaling is inhibited [16]. Currently, we provided evidences that inhibition of Notch signaling partially rescued the angiogenic defects of *maeg* and *itgb1* deficiency embryos, suggesting Maeg-Itgb1 modulates angiogenesis upstream of Notch signaling (Figure 8). Taken together, these observations suggested that EGF-like proteins negatively regulate Notch signaling in a number of biological processes including angiogenesis.

We showed that *maeg* overexpression caused excessive branching of ISVs. In the hyperbranched sprouts, the tip cells project additional filopodia extensions and form connections with sprouts from adjacent ISVs and DLAVs. Most of these connections are lumenized, perfused and are not pruned, suggesting that the aberrant branches develop into functional vessels. Although Maeg overexpression caused hyperangiogenic behaviors of ECs and excessive branching, these embryos did not exactly recapitulate the phenotypes associated with Notch loss-of-function. In *dll4* inactivation embryos, the ectopic branches are largely unperfused, whereas the lumen diameter of the branches in *maeg* gain-of-function embryos is sufficiently large to allow the blood cell perfusion. Furthermore, the locations of the aberrant branches in *maeg* gain-of-function and *dll4* loss-of-function embryos are different. The aberrant branches in *dll4* loss-of-function embryos are mostly restricted in the top position of ISV and DLAV, whereas those ectopic branches widely locate in the dorsal half of ISV. Additionally, inhibition of Notch signaling in *maeg* deficiency embryos partially rescued the angiogenetic defects. These results suggest that *maeg* promotes angiogenesis via additional signaling pathway more than Notch.

## MATERIALS AND METHODS

### Ethics Statement

All animal experimentation was carried out in accordance with the NIH Guidelines for the care and use of laboratory animals (http://oacu.od.nih.gov/regs/index.htm) and ethically approved by the Administration Committee of Experimental Animals, Jiangsu Province, China (Approval ID: SYXK (SU) 2007–0021).

### Zebrafish strains and breeding

Zebrafish embryos and adult were raised and maintained under the conditions as we previously described [38-40]. AB and transgenic zebrafish lines: *Tg(fli1a:nEGFP)*, *Tg(kdrl:EGFP)*, *Tg(kdrl:ras-mCherry)* and *Tg(huC:EGFP)* were used as described in our previous work [39-42]. Embryos were obtained and treated like we previously did [42].

### TALEN construction and microinjection

We designed a pair of TALENs targeting the first exon of Maeg using online tools TALE-NT (https://tale-nt.cac.cornell.edu/) [43]. Left arm and Right arm of Maeg TALEN use FokI heterodimers. The expression plasmids of the TALENs were constructed and linearized with NotI enzyme. TALEN mRNAs were synthesized *in vitro* using the linearized constructs as templates with SP6 mMESSAGE mMACHINE Kit (Ambion), purified with RNeasy Mini Kit (Qiagen), and dissolved in RNase free Ultrapure water (Life Technologies). Equal amounts (100 ng/μl) of Left and Right TALEN mRNA were injected together into the cytoplasm of 1-cell stage zebrafish embryos.

### TALEN *in vivo* activity assay and identification of maeg mutants

The TALEN mRNA injected embryos were maintained in E3 medium (5mM NaCl, 0.17mM KCl, 0.33mM CaCl2, 0.33mM MgSO4) at 28.5 °C. 20 embryos at 24hpf after TALEN mRNA injection were collected and their genomic DNA were extracted and subjected to perform high-resolution melting assay (HRMA) [44]. Primers are listed in supplemental data (Supplementary Table S1). The HRM result shows *maeg* TALEN pair is functional since the melting curve for TALEN mRNA injected embryos is shifted when WT is compared. To identify germ line-transmitted mutations, the microinjected founder (F0) embryos were raised to adulthood. The F0 fish were then outcrossed with wild-type zebrafish to produce F1. 16 of F1 embryos at 24 hpf were collected for genomic DNA extraction respectively. Subsequently, the genomic DNAs were subjected to perform HRMA. Siblings of the F1 embryos that potentially carry heritable mutations were raised to adulthood and individual F1 mutants were identified via PCR amplification and sequencing with fin-clipped DNAs. The primers for genotyping are listed in supplemental data (Supplementary Table S1).

### Injection of morpholinos and mRNAs

Morpholino antisense oligos (MOs; Gene Tools) were prepared at a stock concentration of 1 mM according to the manufacturer's instruction. MOs were diluted to 0.3mM and injected into one-cell stage embryos. MOs for targeting zebrafish *maeg* and *dll4* were the same as previously described [22, 40]. The sequence of standard control MO and *itgb1a* (ENSDART00000039700) translation-blocking MO are listed in supplemental data (Table S1). Zebrafish *maeg* and *mCherry* coding sequence were cloned into PCS2+ vector. The vector template was linearized with NotI Restriction Enzyme (NEB). Sense-capped mRNAs were synthesized with SP6 mMESSAGE mMACHINE Kit (Ambion), purified with RNeasy Mini Kit (Qiagen), and dissolved in RNase free Ultrapure Water (Life Technologies). 2nl *maeg* and *mCherry* mixture (1:1) was injected at 100ng/ μl into cytoplasm of 1/2-cell stage zebrafish embryos.

### RNA extraction, reverse transcription, and PCR

Tissue was homogenized and frozen in TRIzol Reagent (Invitrogen) and stored at -80 °C. The RNA was extracted following the manufacturer's instruction. 1 μg of total RNA was reverse transcribed into cDNA by the use of Transcriptor First Strand cDNA Synthesis Kit (Roche) according to the manufacturer's instructions. Synthesized cDNA was stored at -20 °C. All PCR amplifications were carried out in a total volume of 50μl using specific primers and Advantage 2 Polymerase Kit (Clontech). The primers for PCR are listed in supplemental data (Supplementary Table S1).

### Whole-mount *in situ* hybridization, western blot and immunohistochemistry

Whole-mount *in situ* hybridization with antisense RNA probes was performed according to the previous description [38, 40]. The detailed information of these probes and the primers for generating these probes are listed in supplemental data (Table S1) or described in previous work [22, 30, 40]. DIG-labeled RNA sense and antisense probes were made from the linearized plasmids according to the manufacturer's protocol using the DIG RNA Labeling Kit (SP6/T7) (Roche). The polyclonal antibody against zebrafish Maeg was custom designed and made mouse monoclonal to zebrafish Maeg as previous description [22]. The primary antibody to stain somite boundaries was Thbs4b antibody (GTX129646) bought from (GeneTex). P44/42 MAPK (Erk1/2) (137F5) Rabbit mAb (#4695), Phospho-p44/42 MAPK (Erk1/2) (Thr202/Tyr204) (197G2) Rabbit mAb (#4377), and Phospho-Akt (Ser473) (D9E) XP® Rabbit mAb (#4060) were bought from (Cell Signaling Technology). Rabbit polyclonal AKT Antibody (10176-2-AP) was from (Proteintech Group). The second antibodies used in immunohistochemistry were Goat Anti-Rabbit IgG H&L (TRITC) (ab6718) from (Abcam) and CF594 Donkey Anti-Rabbit IgG (H+L) from (Biotium). Monoclonal Anti-Tubulin (Acetylated antibody produced in mouse) was bought from Sigma (T6793). Immunofluorescence staining and western blot was performed as described previously [40, 42].

### Drug treatment

DAPT was purchased from (Sigma-Aldrich) and dissolved in DMSO. DAPT was used at a final concentration of 100 μM diluted as our pervious description [40]. U0126 was dissolved according to supplier's information (10 mM in DMSO). Up to 60 embryos were treated in 12-well plates U0126 (Sigma) diluted from stock in E3 medium with the concentration of 20μM. PD98059 MAP Kinase Inhibitor (Selleck Chemicals) was used at final concentration at 20μM. E3 medium containing DMSO alone was utilized as control to ensure no effect of the high DMSO treatment.

### Histology and microscopy imaging

Section of the whole-mount *in situ*-hybridized embryos was performed according to the previous description [38]. The results of *in situ* hybridization and photos in bright field were documented with an Olympus DP71 camera on an Olympus stereomicroscope MVX10, Leica imaging system on a Leica compound microscope and Zeiss SteREO Discovery V20 microscope with a Zeiss AxioCam HRc camera. For confocal imaging of blood vessel development in zebrafish embryos, they were anesthetized with egg water/0.16 mg/mL tricaine/1% 1- phenyl-2-thiourea (Sigma) and embedded in 0.6% low melting agarose. Confocal imaging was performed with a Leica TCS-SP5 LSM. Analysis was performed using Imaris software.

### Statistics

Statistical analysis was performed using GraphPad Prism® version 6.0c. One-Way ANOVA (Dunnett test, Tukey test), Fisher's exact test, Student's t-test, Mann Whitney U-test, and χ^2^ test were used (*P*<0.05). When we did the One-Way ANOVA for multiple comparisons test, we chose correction for multiple comparisons.

## SUPPLEMENTARY MATERIALS FIGURES AND TABLE


