# Supplementary material for: The somite-secreted factor Maeg promotes zebrafish embryonic angiogenesis
Source: Oncotarget. 2016 Oct 21;7(47):77749–63. doi: 10.18632/oncotarget.12793 (PMC5363618; doi:10.18632/oncotarget.12793)
Supplement: Supplementary file 1 [file oncotarget-07-77749-s001.pdf]

The somite-secreted factor Maeg promotes zebrafish embryonic angiogenesis

SUPPLEMENTARY FIGURES AND TABLE

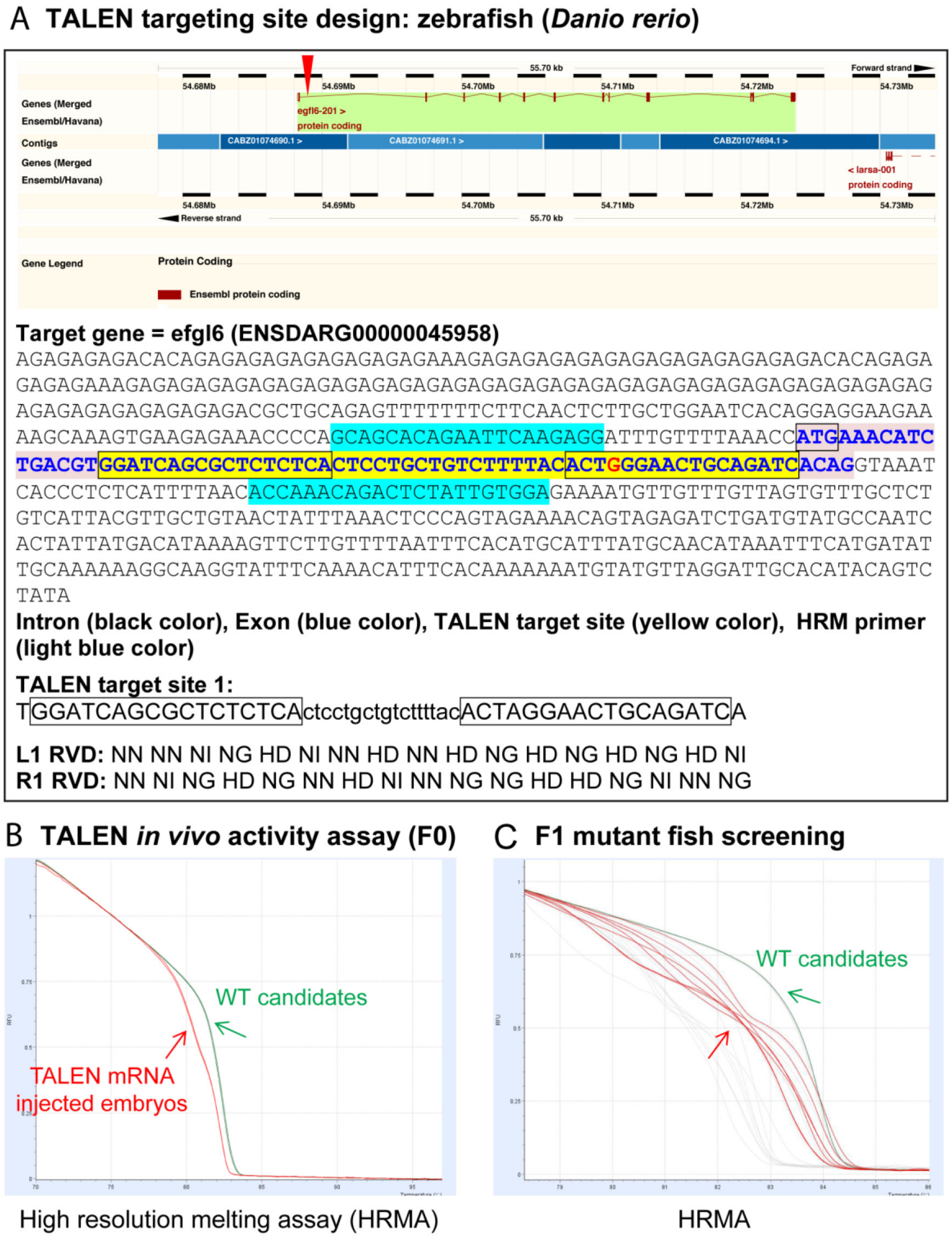

Supplementary Figure S1: Maeg TALEN targeting site design and mutants screening. A. TALEN was designed to target at first exon of *maeg* gene. Red arrowhead indicates the targeting site in zebrafish Genome. Intron is shown in black letters. Exon is shown in blue letters. The target sequence is highlighted in yellow. High-resolution melting assay (HRMA) primers are highlighted in light blue. **B.** The HRMA result shows *maeg* TALEN pair is functional since the melting curve (in red) for TALEN mRNA injected embryos is shifted when WT (in green) is compared. **C.** The genomic DNA of F1 fishes aged at 3 months were collected respectively through fin clip and subjected to perform HRMA. The result shows several F1 fishes are potential mutants (melting curves in red) is shifted, and others are WT (melting curves in green color).

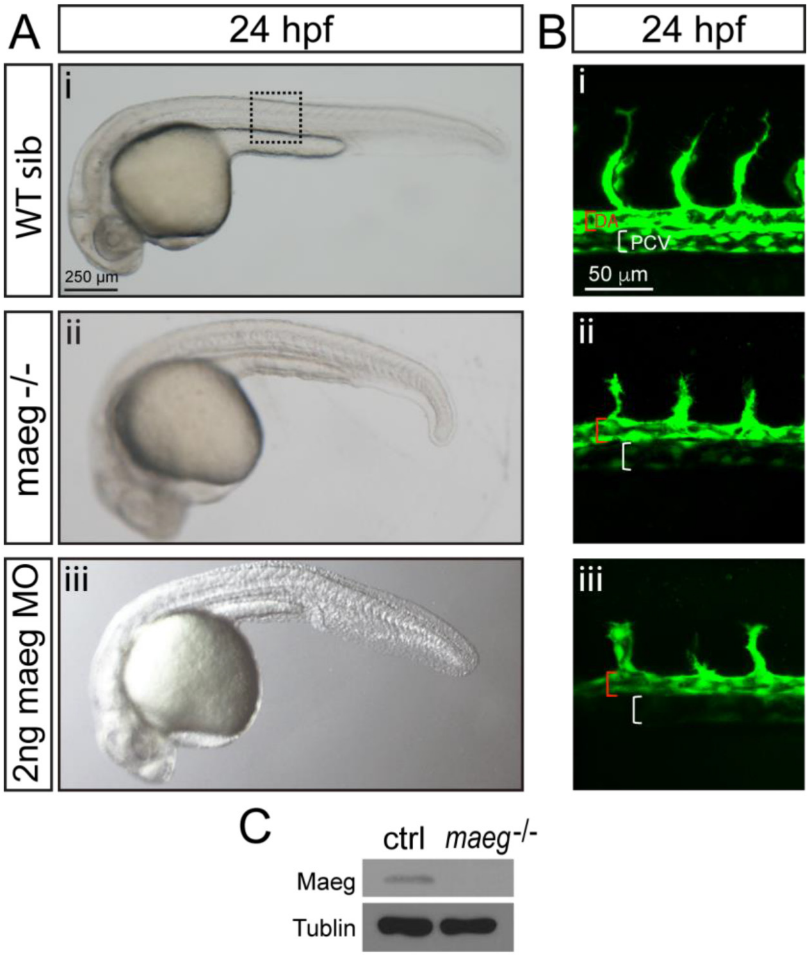

Supplementary Figure S2: *maeg* loss of function results in the blood vessel morphogenesis defects in zebrafish embryos. A. Zebrafish embryos of WT, *maeg*$^{-/-}$ and *maeg* morphants at 24 hpf imaged in bright field. Square in dash line indicates the confocal imaging region of panel B. **B.** Confocal imaging analysis of trunk vascular morphology in WT, *maeg*$^{-/-}$ and *maeg* morphants *Tg(kdrl:EGFP)* embryos at 24 hpf. Red and white square brackets indicate the lumen of the DA and PCV, respectively. DA, dorsal aorta; PCV, posterior cardinal vein. **C.** Western blot analysis of Maeg expression in 24hpf control and mutants whole embryos.

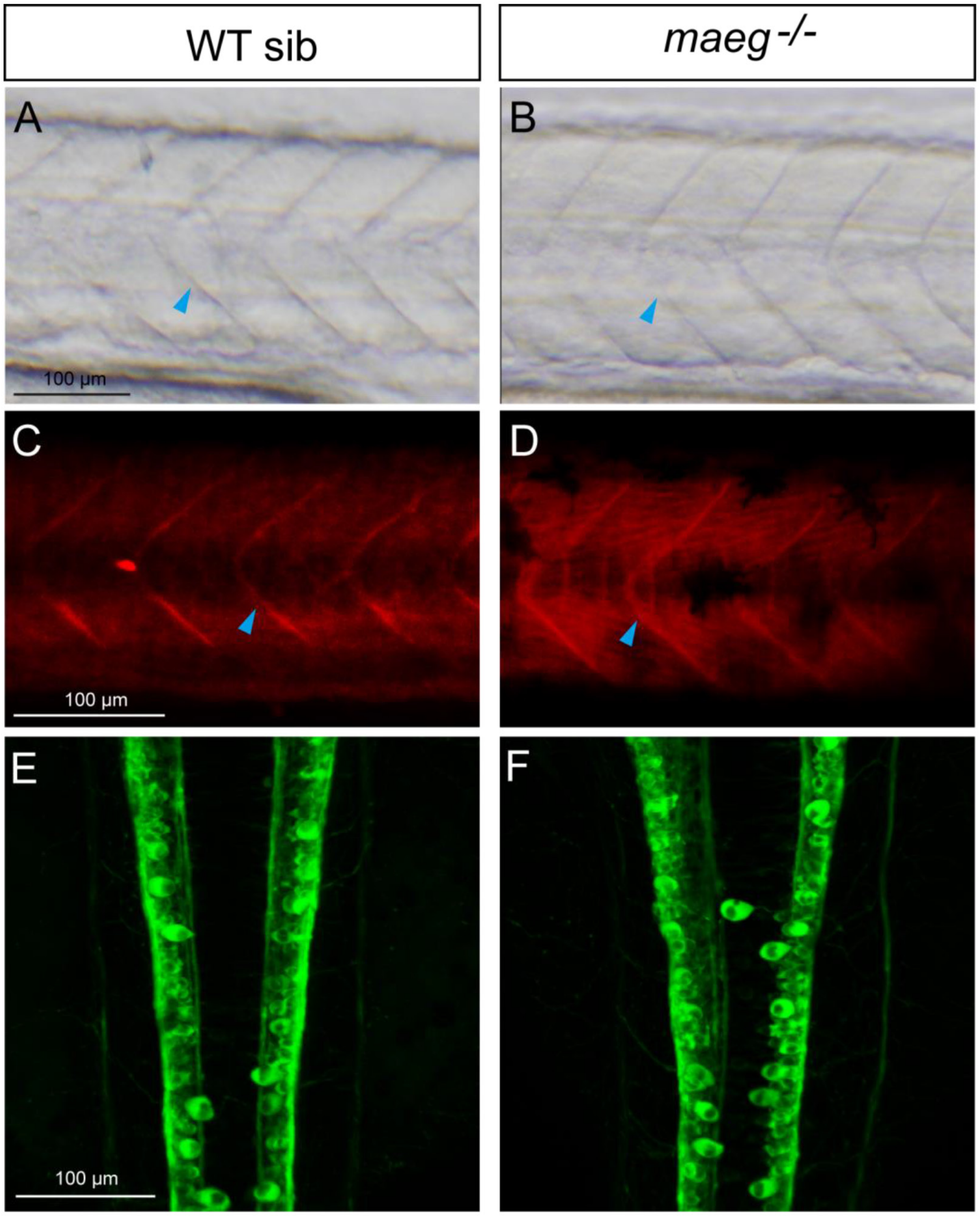

Supplementary Figure S3: Maeg loss-of-function does not affect somite and hindbrain formation. A, B. Microscopy analysis of the somites of zebrafish embryos in brightield. **C, D.** Antibody immunostaining analysis showed the somite boundary in WT and *maeg* mutants. **E, F.** Confocal imaging analysis of hind brain development in WT and *maeg* mutants using *Tg(huC:egfp)* transgenic line.

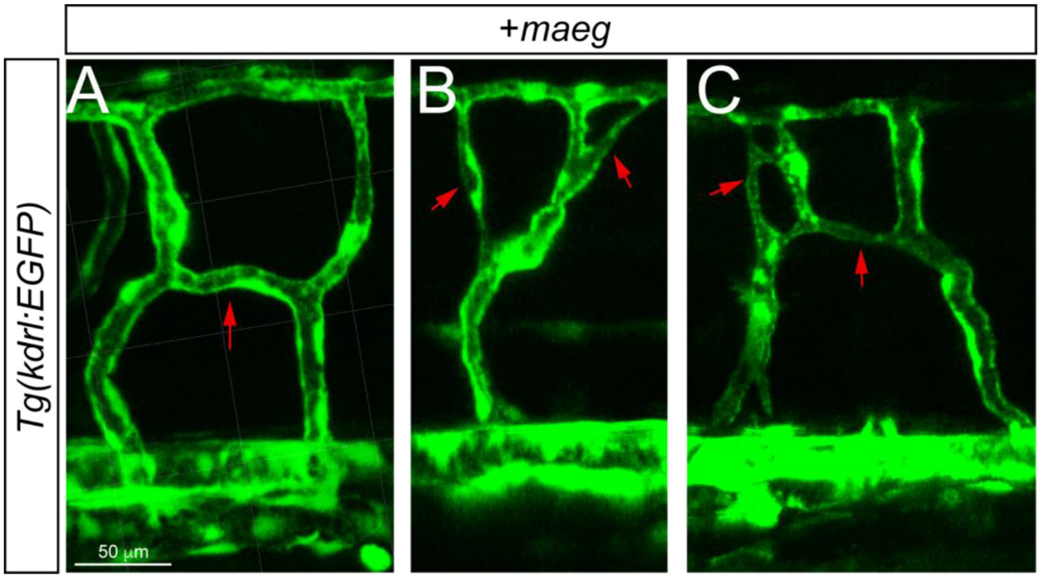

Supplementary Figure S4: Maeg overexpression resulted in the formation of excessive branching. Confocal imaging analysis of ISVs morphology in control and *maeg* mRNA injected *Tg(kdrl:EGFP)* embryos at 48 hpf. Red arrows indicate aberrant structures.

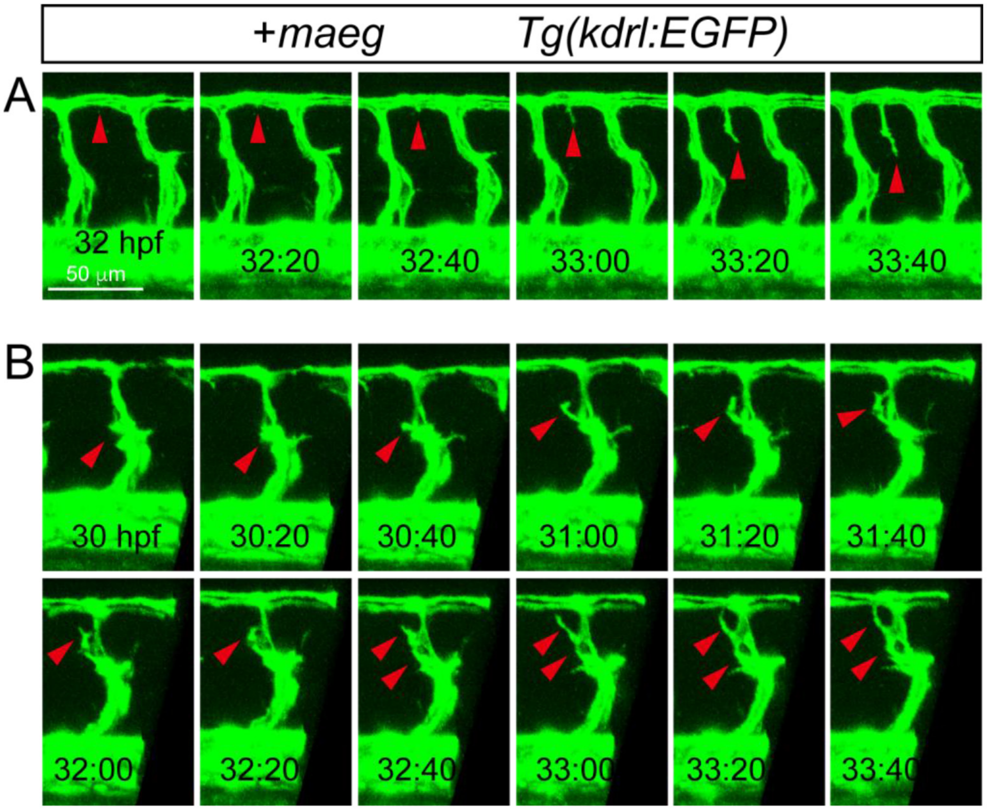

Supplementary Figure S5: The *maeg* gain-of-function embryos resulted in the ectopic branching angiogenic behaviors in the DLAV and ISV. A, B. Still images from *in vivo* time-lapse imaging analysis of the ectopic branching angiogenic behaviors in DLAV and ISV using *Tg(kdrl:EGFP)* embryos. Time (hpf) is noted in the bottom. Red arrowheads indicate the branching sprouts.

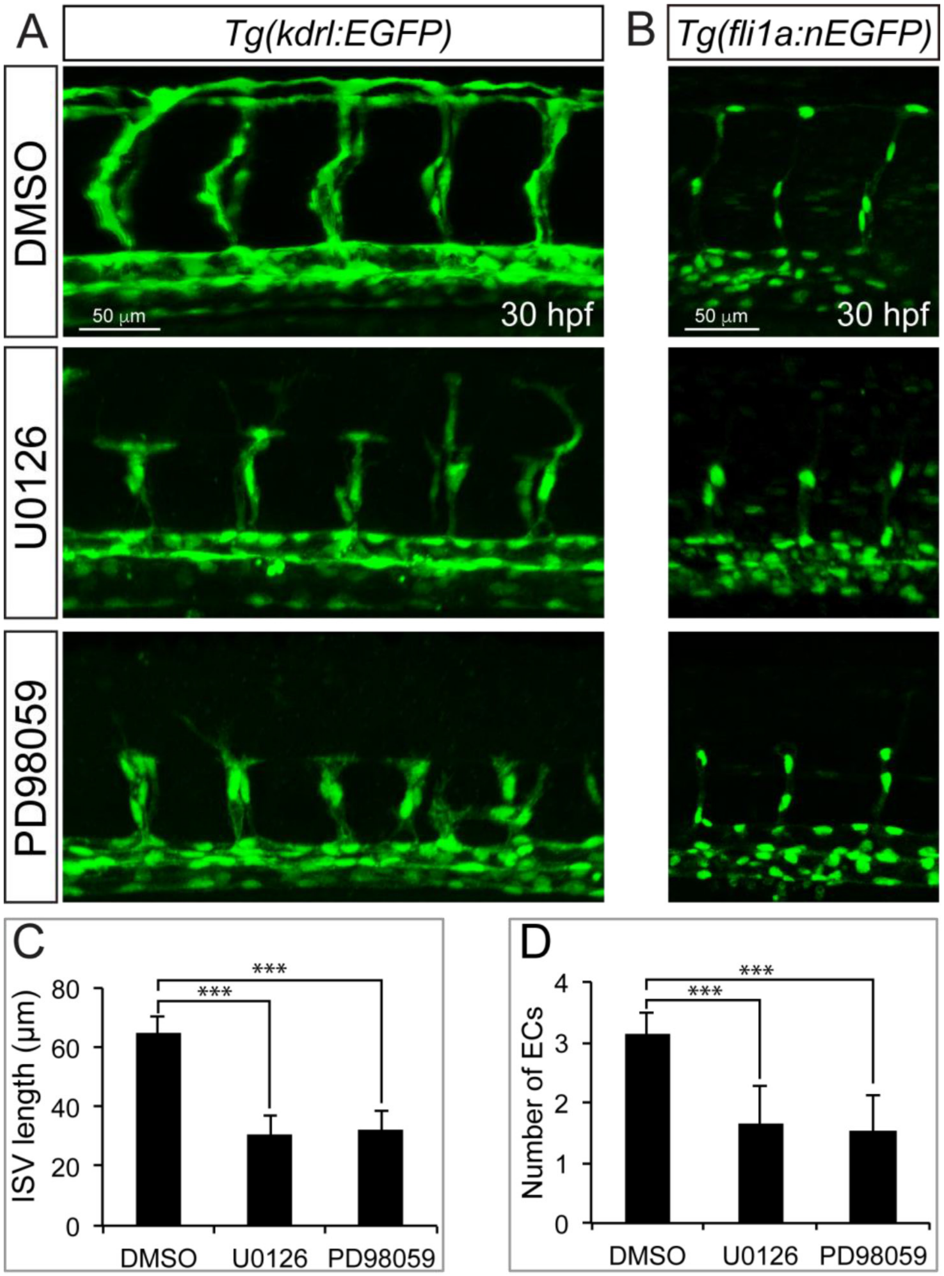

Supplementary Figure S6: Blocking the function of MEK with specific inhibitor U0126 or PD98059 treatment resulted in sprouting angiogenesis defects. A. Confocal images of ISVs morphology in 30 hpf *Tg(kdrl:EGFP)* transgenic embryos treated with DMSO, U0126 or PD98059. **B.** Confocal images of 30hpf *Tg(fli1a:EGFP)* transgenic embryos treated with DMSO, U0126 or PD98059. **C.** The statistics of ISV length in embryos treated with DMSO, U0126 or PD98059. One-Way ANOVA; ***,P<0.001. **D.** Quantification of ECs nuclei number in ISV. Measurements were made from three adjacent ISVs (over yolk) per embryo from 3 independent experiments. One-Way ANOVA; ***, P<0.001.

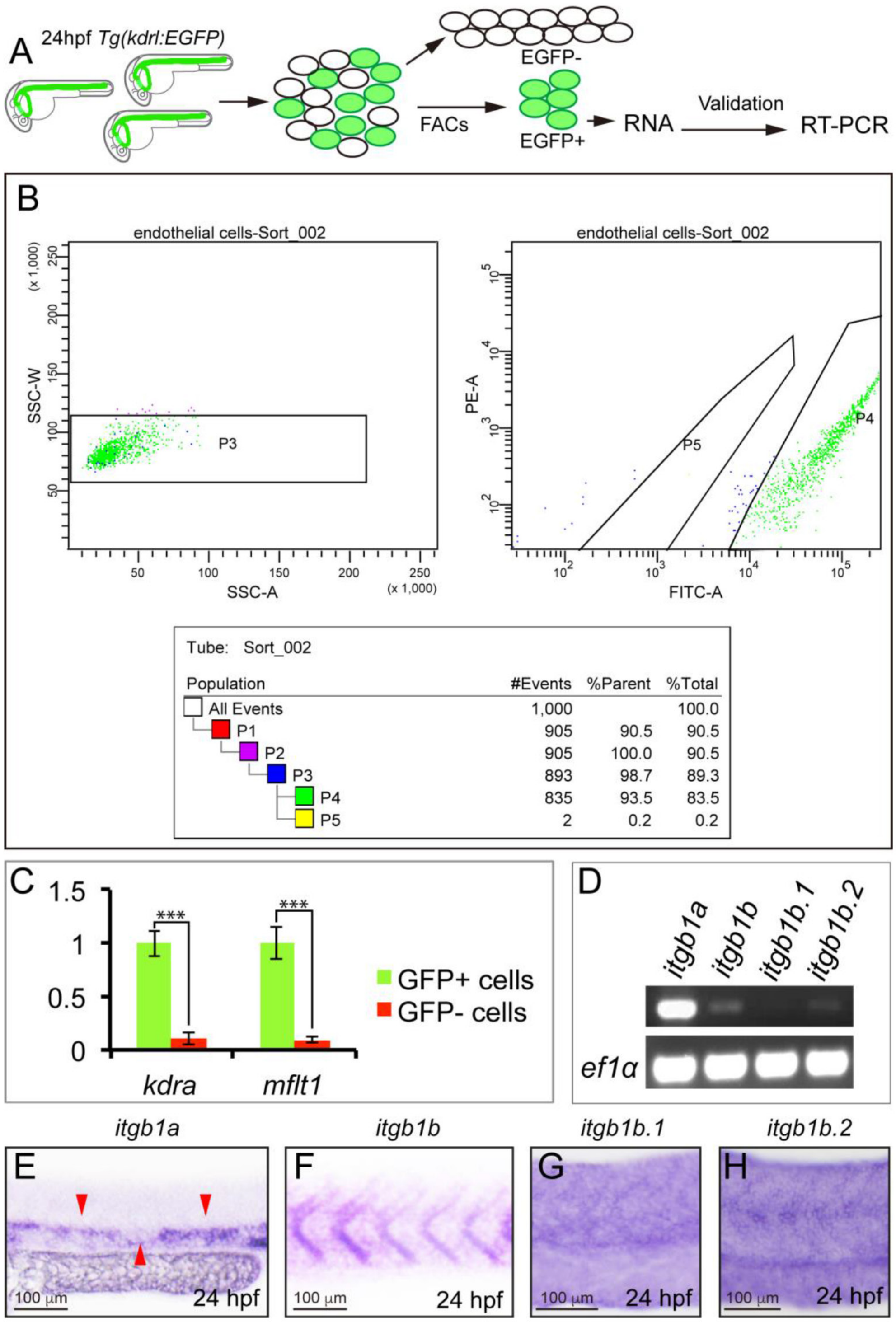

Supplementary Figure S7: Identification of *itgb1* isoform highly enriched in zebrafish ECs. A. Diagram of the experimental procedure. **B.** Diagnostic FACS analysis of the sorted cells. P4 is the GFP positive cells; P5 is GFP negative cells. **C.** Quantitative PCR analysis of *kdra* and *mflt1* gene expression between GFP positive cells and GFP negative cells after FACs sorting. **D.** RT-PCR analysis of expression of *itgb1a*, *itgb1b*, *itgb1b.1* and *itgb1b.2*. **E-H.** Whole mount *in situ* analysis of *itgb1a*, *itgb1b*, *itgb1b.1* and *itgb1b.2* at 24 hpf.

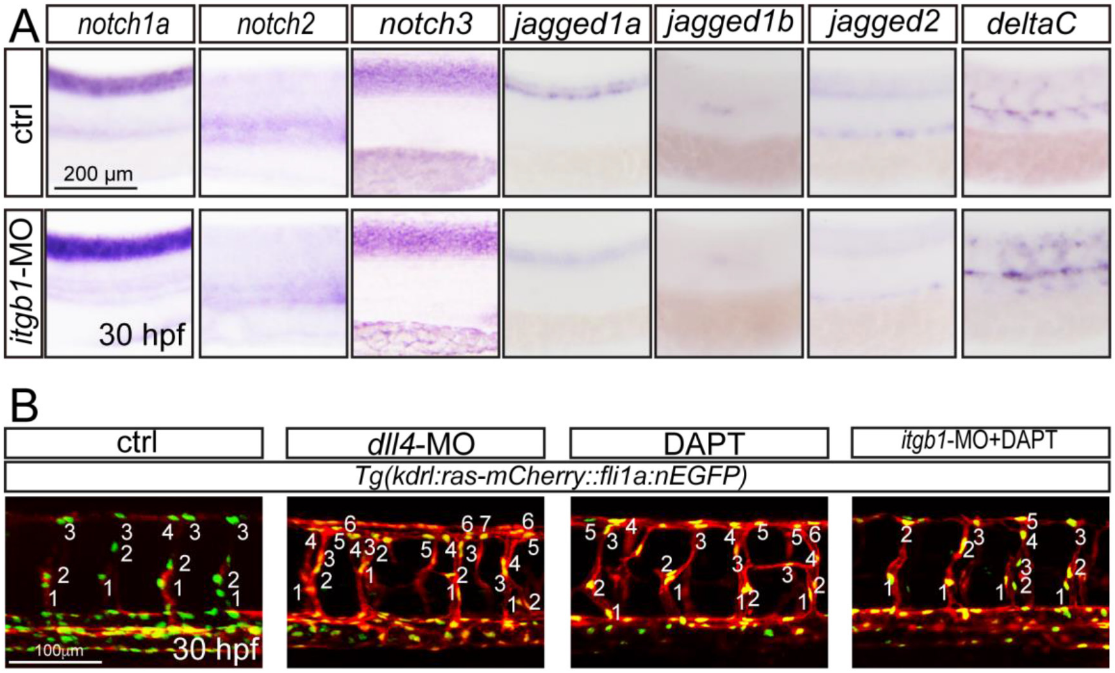

Supplementary Figure S8: The phenotype of *itgb1* loss-of-function involves Notch signaling. A. Whole mount in situ hybridization analysis of zebrafish embryos using antisense *notch1a*, *notch2*, *notch3*, *jagged1a*, *jagged1b*, *jagged2* and *deltaC* probes. 30 hpf, lateral view. **B.** Confocal images of ISVs in control embryos, *dll4* morphants, control embryos treated with DAPT, and *itgb1* morphants treated with DAPT using *Tg(kdrl:ras-mCherry::fli1a:nEGFP)* transgenic line.

Supplementary Table S1: Sequence information of primers and morpholinos used.

	Primers for RT-PCR	NCBI Accession	Forward (5'-3')	Reverse (5'-3')
1	*ef1a*-1	NM_131263	TGATCTACAAATGCGGTGGA	CAATGGTGATACCACGCTCA
2	*ef1a*-2	NM_131263	CTTCAACGCTCAGGTCATCA	CGGTCGATCTTCTCCTTGAG
3	*itgb1a*	NM_001034971.1	ACATACGCCGTCAACAACAA	GAGAGATTGCGTGAATGTGC
4	*itgb1b*	NM_001034987.1	GCATTGGCAGAGTTTGTGAA	TCGCACGTGTCTTTCTTGTC
5	*itgb1b.1*	NM_001034979.1	GTGATTGGCTGGAGGAATGT	CAGCCTTTAGCCGTGATTTC
6	*itgb1b.2*	NM_212928.1	TGACAAGAACAAACCCGTCA	ACATTCCTCCAGCCAATCAC
7	*maeg*-HRMA	NM_001002457	GCAGCACAGAATTCAAGAGG	TCCACAATAGAGTCTGTTTGGT
8	maeg-genotyping primer		GCAGCACAGAATTCAAGAGG	TCCACAATAGAGTCTGTTTGGT

	Sequences of morpholinos
standard control	5'-CTCTTACCTCAGTTACAATTTATA-3'
itgb1a translation-blocking MO	5'- TATGAAAAGTAGCTTCAGGTCCATC-3'

> Maeg coding sequence(RGD→RGE)

Atgaaacatctgacgtggatcagcgctctctcactcctgctgtcttttacactaggaactgcagatcacagacagcggcggcagatctccgtcatgagtgggctgggtgtgtgtcga
tacggcagccggttggagtgctgctacggctggaagaaaaacactaaaggacaatgtgaagctcagtgtgatctgggctgcaaacacggcgagtgtgtcggccccaacaaatg
caagtgttttcctggatacactggaaagacctgcagccaagatctgaatgagtgtggtctgaagcctcgtccctgtgagcatcgctgtatgaacacattcggcagctatatgtgctact
gcttaaacggctacatgctaatgcctgatggatcctgtgcaaactccaggacgtgctctctggctcactgtcagtacggctgtgaggaggtgcagtctgaggttcgctgtctctgtcc
atcccctggccttcagctcgggtctgatggaaagacctgcgaggacattgatgaatgtgcgactgggaaaaaccagtgtccgtttaaccggcagtgcacaaacacattcggcagct
actactgcaagtgtcagccaggatacgacctcaaatacatcaatggaaaatacgactgcgttgatgtaaatgagtgcacgtcgaacacacacaagtgcagtcatcacgctgaatgc
atcaacactcttgggtcctacaagtgtaaatgcaagcagggcttccgtggcagcggattcgattgttcagtcaagccattttatcacagatcctgggacagcagtaaaggaaacaca
gatgtgcttgtgaacgcgattcccgattttccgtccaaaacccggattttgggtaaatttgagaacagcatcccagagcctgtgctgactgaatctcctcgacagcatctgcaaccctt
cgattatgatggagaggtttacatcggcgctccacaggaaacaccagtgtttcctgaggaagaagaagtggaagaggaagaggaagaggacacagagaaccagctggaggat
gaagagctgaccagcagaggagaa(RGE)gtgttttctcaggatgattttgtgtctttttatcaatcagagttagagttgaaggagatccaggcggcgccgctgaaggaaagatttct
gacggattgtgactttgaccgcggtgcttgtgaatgggttcaggataacgcagatgatctggactggaccatcaaatatcatccagacgggcgggagtattacctggctctcgaggg
ccccgtggggaacaggcgtgagcaggcccgcatgaagctcctgctggaagatcacatgcggcagagcagcttctgtctgaggtttgatttccgcattgagggtccgcagacgggt
gttttacgggtgaaactggacaacagcgccatctccatctgggagagaaaacagagtcagaagcagagctggcagagcgagcagatcagcatcagctggacagacagtaatcct
gaagcggtggtctttgaggctgagcggggacgctccggcactggagaaatcgctctggatcatgtgctgctgatctctggagaatgttcagaagataaaacactcgacttctag
